# 

**Published:** 1887-12

**Authors:** 


RECEIPTED.—1887—Drs. T. B. Robertson, C. M. Bold, J. C. Moody, to July; H. Mobley, to March; J. A. Daniel, W. Brewington, W. S. Bradford, G. W. Bowling, J. F. Burnett, J. F. Davis. 1888—J. B. Long, to October; P. M. Catching, to July; M. M. Evans, to July; J. H. Wysong, to July.

